# Attitudes Toward Gender-Based Violence Among Sexually Active Adult Men at High Risk for HIV in Rustenburg, South Africa

**DOI:** 10.1177/15579883221106331

**Published:** 2022-06-24

**Authors:** Heeran Makkan, Pholo Maenetje, Candice M. Chetty-Makkan, Evans Muchiri, Mary H. Latka, Vinodh A. Edward, Matt A. Price, Gloria Omosa-Manyonyi, Christina Lindan

**Affiliations:** 1 Rustenburg Research Centre, The Aurum Institute, Rustenburg, South Africa; 2 Department of Interdisciplinary Social Science, Public Health, Utrecht University, Utrecht, The Netherlands; 3 School of Pathology, Faculty of Health Sciences, University of the Witwatersrand, Johannesburg, South Africa; 4 Health Economics and Epidemiology Research Office, Faculty of Health Sciences, University of the Witwatersrand, Johannesburg, South Africa; 5 Department of Environmental Health Sciences, Yale School of Public Health, New Haven, CT, USA; 6 School of Health Sciences, College of Health Sciences, University of KwaZulu-Natal, Durban, South Africa; 7 Department of Epidemiology and Biostatistics, University of California, San Francisco, San Francisco, CA, USA; 8 IAVI, New York, NY, USA; 9 School of Medicine, College of Health Sciences, University of Nairobi, Nairobi, Kenya

**Keywords:** gender-based violence (GBV), sexual behavior, HIV, men, sexually transmitted infection (STI), HIV prevention, South Africa

## Abstract

Gender-based violence (GBV) toward women is widespread and has been associated with increased HIV risk. We investigated attitudes toward GBV among men living in Rustenburg, South Africa, who were enrolled in a longitudinal HIV incidence study. Participants were 18 to 49 years old, reported high risk sexual activity in the last 3 months, and were HIV-uninfected. Attitudes toward GBV were evaluated using responses to a five-item standardized questionnaire about men perpetrating physical violence on a female spouse; responses to each item were scaled from 1 (*no agreement*) to 4 (*strong agreement*) and summed. Total scores >10 were considered permissive toward GBV. Among the 535 men analyzed, nearly half (*N* = 229, 42.8%) had a GBV score >10. Being young (18–24 years) (adjusted odds ratio [aOR] = 1.53, 95% confidence interval [CI] [1.06, 2.22]), having less years of education (aOR = 1.61, 95% CI [1.11, 2.32]), and reporting no current sexual partner at baseline (aOR = 2.10, 95% CI [1.06, 4.14]) were independently associated with permissive attitudes toward GBV. The following behaviors reported in the last 3 months were also associated with high GBV scores: having a new female partner (aOR = 1.78, 95% CI [1.02, 3.10]), and having had an STI (aOR = 1.85, 95% CI [1.15, 2.99]). Consuming alcohol prior to sex in the last month (aOR = 1.59, 95% CI [1.09, 2.31]) was also associated with high GBV scores. A large proportion of South African HIV-uninfected men in this analysis reported permissive attitudes toward GBV. These attitudes were associated with HIV risk behavior. Integrating GBV and HIV prevention programs is essential.

## Introduction

South Africa has the highest burden of HIV infection of any country in the world. In 2019, an estimated 7.5 million people were living with HIV in South Africa (UNAIDS, 2020a, 2020b). The HIV incidence is highest among adolescent girls and young women aged 15 to 24 years, many of whom are affected by gender-inequalities and have acquired HIV through sexual relationships with older men (Simbayi et al., 2019; UNAIDS, 2016). Behaviors associated with becoming HIV infected include having multiple sexual partners, engaging in transactional sex, having sex without condoms, early sexual debut, not knowing one’s HIV status, alcohol use, and illicit drug use. Women who are exposed to gender-based violence (GBV) are also more likely to be at risk for HIV acquisition (Li et al., 2014; Rigby & Johnson, 2017; UNAIDS, 2016; World Health Organization, 2014).

The World Health Organization identified GBV as a human rights and public health concern that affects up to 30% of women worldwide (Li et al., 2014; Rigby & Johnson, 2017a; UNAIDS, 2016; Watt et al., 2017; World Health Organization, 2014). GBV includes sexual, physical, psychological or emotional violence, or economic abuse. A 2016 South African Demographic and Health Survey reported that one in four women older than 18 years of age experienced some form of GBV in their lifetime; women in the survey were more likely to report physical (21%) compared with emotional (17%) or sexual violence (6%) (National Department of Health [NDoH] et al., 2019). Women who were at risk of HIV and women who suffered from GBV had similar characteristics, such as having multiple partners, engaging in transactional sex, and being in age-disparate relationships. In addition, women who are survivors of GBV were less likely to negotiate condom use, test for HIV, or disclose their HIV status to partners (World Health Organization, 2000, 2014). GBV, when involving physical rape, increases the risk of HIV transmission and sexually transmitted infections (STI), including by causing vaginal injury (Dunkle & Decker, 2013; World Health Organization, 2004).

Among men, characteristics associated with HIV risk behavior and with perpetrating GBV are also similar. One survey reported that HIV-infected South African men compared with HIV-uninfected men were less likely to disclose their HIV status to their partners or practice safe sex and were more likely to have multiple young and financially dependent sexual partners (Simbayi et al., 2019; UNAIDS, 2016). These characteristics have also been associated with men who perpetrate GBV toward women (Fleming et al., 2019). Perpetrators of GBV were disinclined to test for HIV, had low levels of HIV knowledge, and feared being stigmatized if they accessed HIV treatment and care (Fleming et al., 2019).

Because of the relationship between HIV risk and GBV, identifying men who engage in GBV and involving them in gender-equality interventions could reduce both GBV and HIV transmission, especially among communities most affected by HIV (Jewkes et al., 2006, 2015; Lansford et al., 2014; Townsend et al., 2013). Screening men for their attitudes toward GBV could be helpful. A systematic review of GBV reported that men who expressed attitudes that favored violence toward women and male sexual entitlement were more likely to perpetrate GBV (McCarthy et al., 2018). Interventions that fostered belief in gender-equality among men reduced violent behavior (Li et al., 2014; Zembe et al., 2015).

During the current COVID-19 pandemic, there have been increased reports of domestic violence in South Africa, which could be due to being confined to home, with less access to exercise, work, friends, and sporting events (Glover et al., 2020; Joska et al., 2020). The number of sexual offenses and rape cases reported to the South African Police Service in 2019 were 52,420 and 41,583, respectively (DWYPD, 2020). In contrast, within only the first week of South Africa’s COVID-19 lockdown in 2020, 87,000 cases of domestic and interpersonal violence were filed (Joska et al., 2020), and included women being murdered by their spouse or sexual partner (ANA Reporter, 2020; Ndaba, 2020). The increase in GBV was likely exacerbated in the shift of resources to respond to the COVID-19 pandemic, and a decrease in GBV prevention and response services (Roy et al., 2022). Increase in reports of GBV have raised public awareness of the scope of GBV experienced by women in South Africa, and invigorated efforts to promote belief in gender-equality among adolescent boys and young men (Glover et al., 2020; Joska et al., 2020).

Despite the similarity in the factors associated with HIV transmission risk and with GBV, few studies have focused on attitudes toward GBV among men at high risk for HIV. We therefore performed a secondary data analysis to evaluate the association between attitudes toward GBV and self-reported HIV risk behavior among men entering an HIV vaccine preparedness study in Rustenburg, South Africa. We hypothesized that men with permissive attitudes toward GBV would be more likely to report behaviors associated with HIV risk. This information may help inform interventions that simultaneously target high-risk sexual behavior and GBV.

## Method

### Study Design and Setting

We performed a secondary data analysis of baseline data collected from a longitudinal cohort study designed to measure HIV incidence and sexual behavior among adult men from Rustenburg, South Africa (Maenetje et al., 2019). Rustenburg is a city of approximately 550,000 in the North West province of the country and has an economy mainly driven by the platinum mining industry (Massyn et al., 2020). It is one of the fastest growing cities in South Africa and was chosen as a site to conduct HIV vaccine efficacy trials because of its expanding HIV epidemic. Previous studies reported high HIV incidence rates among women and men who have sex with men (MSM) living in this area (Kamali et al., 2015).

### Study Participants

Study procedures have been described in detail previously (Maenetje et al., 2019). Briefly, men who were 18 to 49 years old, HIV negative, and reported at least one of the following in the last 3 months were enrolled between May 2012 and June 2015: (1) had been treated for, diagnosed with, or had symptoms of an STI, (2) had vaginal and/or anal intercourse with more than one sexual partner, (3) had vaginal and/or anal intercourse with a new sexual partner, or (4) had sex with a partner known to be HIV infected. Men who reported having sex with a female sex partner (including those who reported both male and female partners) were included in this analysis; those who only had sex with other men were excluded.

### Ethical Review

The parent study was approved by the Biomedical Research Ethics Committee of the University of KwaZulu-Natal, and the Research Committee of the Northwest Provincial Department of Health. All participants in the parent study provided written informed consent.

### Measurements

Participants completed interviewer-administered questionnaires (Appendix A and B). The questionnaires evaluated sociodemographics, a participant’s perception of his own financial status and relative to his neighbors, sexual behavior in the last 3 months including number of female sex partners, number of new female sex partners, frequency of condom use with female partners (never, sometimes or always), age of sexual debut, and history of treatment or symptoms of an STI. At baseline, participants were asked whether they currently cohabited with a sexual partner. Participants who had a female partner within the past 3 months prior to screening but not at the baseline visit responded as having “no current partner.” Men were asked about the following behaviors in the last month: how frequently they used alcohol (never, occasionally [1–3 times/month or weekly], or daily), how frequently they consumed alcohol prior to sex (never, sometimes, frequently, always), and whether they had used any illicit drugs.

Attitudes toward GBV were assessed at baseline by asking men to respond to a five-statement questionnaire that was developed and standardized for use in the Multiple Indicator Cluster Survey (MICS) (Cappa, 2014; Lansford et al., 2014). The MICS is a socioeconomic household survey developed by the United Nations Children’s Fund. The GBV questionnaire assesses attitudes toward physical abuse perpetrated by husbands toward a female spouse. The five items are “A husband is justified in hitting or beating his wife if she goes out without telling him,” “A husband is justified in hitting or beating his wife if she neglects the children,” “A husband is justified in hitting or beating his wife if she argues with him,” “A husband is justified in hitting or beating his wife if she refuses to have sex with him,” and “A husband is justified in hitting or beating his wife if she burns the food.” Participants are asked to indicate the extent to which they agreed with each statement: “strongly disagree,” “disagree,” “agree” or “strongly agree.” The GBV questionnaire only assesses attitudes toward physical violence directed at a spouse and does not assess other aspects of GBV such as sexual, psychological, or emotional violence, or economic abuse.

### Data Analyses

Our primary outcome variable was reporting a permissive attitude toward GBV based on responses to the five-item GBV questionnaire. Responses to each statement were scored from 1 (*strongly disagree*) to 4 (*strongly agree*). Scores were summed, with a total range of 5 to 20. We used the median to produce a dichotomous outcome variable; a score of ≤10 was considered indicative of “negative attitudes toward GBV,” and a score of >10 was considered indicative of “permissive attitudes toward GBV” (Lansford et al., 2014).

Data analyses were conducted in Stata v14.0 (Stata Corporation, College Station, Texas, USA). We calculated frequency distributions of categorical variables and means and standard deviations (*SD*) for continuous variables. If continuous variables did not follow the normal distribution, we calculated medians and the interquartile range (IQR). To determine the internal consistency of the GBV scale, we calculated the Cronbach’s alpha. We calculated the proportion of men within each category of covariates who had permissive attitudes toward GBV.

We used univariate logistic regression to evaluate the association of variables with permissive attitudes toward GBV and calculated unadjusted odds ratios (OR) and 95% confidence intervals (CI). Variables significantly associated with the outcome at *p* ≤ .1 by univariate analysis were included in multivariable adjusted models and retained if the *p* < .05. Models also adjusted for number of female partners and knowledge of HIV status of sex partners.

## Results

Among the 718 men who were enrolled in the parent study, we excluded data from 90 (12.5%) because of incomplete baseline data, from 21 (3.0%) who reported having no female partner in the past 3 months, and from 72 (10.0%) who reported not being sexually active in the past 3 months. Thus, for this study, we analyzed baseline data from 535 participants. A little less than half (*n* = 252, 47.1%) were 18 to 24 years old, and the median age was 26.4 years (IQR = 22–29) (Table 1). More than half were unemployed (*N* = 285, 53.4%), and 313 (58.5%) had completed some level of post-secondary school education. In the last 3 months, 286 (53.5%) of men reported having multiple female sexual partners, 82 (15.3%) reported having two or more new female partners, and 332 (62.1%) did not know the HIV status of their partner(s). Only six men (1.1%) reported both male and female partners. Two hundred fourteen (40.0%) men reported consuming alcohol prior to sex in the last month. Ninety men (16.8%) reported a history of being treated for or having symptoms of an STI in the 3 months prior to screening.

**Table 1.** Baseline Characteristics and the Proportion Who Reported Permissive Attitudes Toward Gender-Based Violence (GBV), Among Men in Rustenburg, South Africa (N = 535).

	Total sample		Permissive toward GBV	
Characteristics	*n*	(%)^a^	*n*	(%)^b^
All	535	(100.0)	229	(42.8)
Sociodemographics				
Age, years (median age = 26.4, IQR = 22–29)				
18–24	252	(47.1)	120	(47.6)
25–49	283	(52.9)	109	(38.5)
Employment status				
Unemployed	285	(53.4)	125	(43.9)
Student/learner	41	(7.7)	18	(43.9)
Employed^c^	208	(39.0)	86	(41.3)
Financial status, self-report				
Poor	304	(56.8)	129	(42.4)
Comfortable	231	(43.2)	100	(43.3)
Family financial status compared with other families in neighborhood, self-report				
Poorer	88	(16.5)	39	(44.3)
About the same	365	(68.5)	152	(41.6)
Better	80	(15.0)	38	(47.5)
Cohabiting with sexual partner				
No	423	(79.2)	173	(40.9)
Yes	71	(13.3)	33	(46.5)
No current partner^d^	40	(7.5)	23	(57.5)
Highest level of education				
Some primary or secondary school	222	(41.5)	109	(49.1)
Post-secondary school^e^	313	(58.5)	120	(38.3)
Frequency of alcohol consumption, last month				
None	163	(30.5)	61	(37.4)
Occasional^f^	356	(66.5)	162	(45.5)
Daily	16	(3.0)	6	(37.5)
Illicit drug use, last month				
Yes	134	(25.0)	61	(45.5)
No	401	(75.0)	168	(41.9)
Sexual behavior, last 3 months				
Gender of sex partners				
Female only	529	(98.9)	224	(42.3)
Male and female	6	(1.1)	5	(83.3)
Number of female partner(s)				
1	249	(46.5)	98	(39.4)
≥2	286	(53.5)	131	(45.8)
Frequency of condom use (with female partners only)				
Never	113	(21.1)	50	(44.2)
Sometimes	278	(52.0)	119	(42.8)
Always	144	(26.9)	60	(41.7)
Number of new female partners				
0	290	(54.2)	110	(37.9)
1	163	(30.5)	82	(50.3)
≥2	82	(15.3)	37	(45.1)
Known HIV positive female sex partner(s)				
Yes	13	(2.4)	2	(15.4)
No	190	(35.5)	70	(36.8)
Do not know HIV status	332	(62.1)	157	(47.3)
Other sexual behavior history				
Alcohol consumption prior to sex, last month				
Yes	214	(40.0)	106	(49.5)
No	321	(60.0)	123	(38.3)
Age of sexual debut, years				
≤14	78	(14.6)	35	(44.9)
15–18	356	(66.5)	152	(42.7)
≥19	101	(18.9)	42	(41.6)
History of treatment or symptoms of STI, past 3 months				
Yes	90	(16.8)	49	(54.4)
No	445	(83.2)	180	(40.4)

*Note.* Differences in *N* are due to few missing responses. GBV = gender-based violence; IQR = interquartile range; STI = sexually transmitted infection, self-report of signs/symptoms or having been treated for an STI.

a These are column percentages. ^b^Depicts row percentages of the proportion who were permissive of GBV only. ^c^Includes permanent, temporary, self-employment. ^d^Participants who had a female partner 3 months prior to screening but not at baseline. ^e^Includes apprenticeship or studentships. ^f^1–3 times/month or weekly.

Overall, 229 (42.8%) men were classified as having “permissive attitudes toward GBV” (Table 1). Cronbach’s alpha for the five statements about GBV was 0.85. A higher proportion (*n* = 120, 47.6%) of younger men (18–24 years old) had permissive attitudes toward GBV compared with older men (*n* = 109, 38.5%). Those with less years of schooling were more likely to have permissive attitudes toward GBV (*n* = 109, 49.1%) compared with those with post-secondary school education (*n* = 120, 38.3%). For those who reported multiple new female partners or a new female partner in the past 3 months, 131 (45.8%) and 82 (50.3%), respectively, had permissive attitudes toward GBV, compared with those who had one female partner or no new female partner. A greater proportion of men who reported consuming alcohol before sex were permissive toward GBV (*n* = 106, 49.5%), compared with those who did not combine sex and alcohol (*n* = 123, 38.3%). Most men in the sample (*n* = 332, 62.1%) reported not knowing their partners’ HIV status, among whom 157 (47.3%) reported permissive attitudes toward GBV. Among those who reported an STI (*n* = 90, 16.8%), 49 (54.4%) reported permissive attitudes toward GBV compared with 180 (40.4%) of those who did not. The proportion of men responding to each statement about GBV is shown in detail in Table 2.

**Table 2.** Responses From 535 Men Regarding Their Attitudes Toward GBV Likert-Type Scale Statements.

Statements	Number of respondents:			
	Strongly disagree	Disagree	Agree	Strongly agree
	*n* (%)	*n* (%)	*n* (%)	*n* (%)
A husband is justified in hitting or beating his wife if she goes out without telling him	104 (19.4)	298 (55.7)	124 (23.1)	9 (1.7)
A husband is justified in hitting or beating his wife if she neglects the children	67 (12.5)	271 (50.7)	187 (35.0)	10 (1.9)
A husband is justified in hitting or beating his wife if she argues with him	72 (13.5)	318 (59.4)	138 (25.8)	7 (1.3)
A husband is justified in hitting or beating his wife if she refuses to have sex with him	102 (19.1)	342 (63.9)	80 (15.0)	11 (2.1)
A husband is justified in hitting or beating his wife if she burns the food	96 (17.9)	366 (68.4)	68 (12.7)	5 (0.9)

*Note.* GBV = gender-based violence.

Based on univariate logistic regression, men who were younger, had a lower level of education, had new female sex partners, consumed alcohol prior to sex, reported an STI, and did not report a current sexual partner were more likely to report permissive attitudes toward GBV (Table 3). In multivariable analysis, being 18 to 24 years old (adjusted odds ratio [aOR] = 1.53, 95% CI [1.06, 2.22]), having secondary education or less (aOR = 1.61, 95% CI [1.11, 2.32]), having a new female sex partner in the last 3 months (aOR = 1.78, 95% CI [1.02, 3.10]), consuming alcohol prior to sex in the past month (aOR = 1.59, 95% CI [1.09, 2.31]), reporting an STI (aOR = 1.85, 95% CI [1.15, 2.99]), and not having a sexual partner at baseline (aOR = 2.10, 95% CI [1.06, 4.14]) were independently associated with permissive attitudes toward GBV.

**Table 3.** Factors Associated With Permissive Attitudes Toward Gender-Based Violence Among Men, Rustenburg, South Africa.

Characteristic	Univariate analysis			Multivariable analysis		
	Unadjusted OR	95% CI	*p* value	Adjusted OR	95% CI	*p* value
Age, years						
18–24	1.45	[1.03, 2.05]	.03	1.53	[1.06, 2.22]	.02
25–49	Ref			Ref		
Employment status						
Employed^a^	Ref					
Student/learner	1.11	[0.56, 2.18]	.76			
Unemployed	1.10	[0.77, 1.59]	.58			
Financial status, self-report						
Poor	Ref					
Comfortable	1.04	[0.73, 1.46]	.84			
Self-report of family financial status compared with other families in neighborhood						
Poorer than others	Ref					
About the same	0.90	[0.56, 1.43]	.65			
Better	1.14	[0.62, 2.09]	.68			
Cohabiting with sexual partner						
No	Ref					
Yes	1.25	[0.76, 2.08]	.38	1.54	[0.89, 2.69]	.12
No current partner^b^	1.96	[1.01, 3.77]	.05	2.10	[1.06, 4.14]	.03
Highest level of education						
Post-secondary school^c^	Ref			Ref		
Some primary or secondary school	1.55	[1.10, 2.20]	.01	1.61	[1.11, 2.32]	.01
Frequency of alcohol consumption, last month						
None	Ref					
Occasional^d^	1.40	[0.96, 2.04]	.09			
Daily	1.00	[0.35, 2.90]	.99			
Illicit drug use, last month						
No	Ref					
Yes	1.16	[0.78, 1.72]	.46			
Number of female partner(s), last 3 months						
1	Ref			Ref		
≥2	1.30	[0.92, 1.84]	.13	0.71	[0.41, 1.22]	.21
Frequency of condom use (with female partners only), last 3 months						
Never	Ref					
Sometimes	0.94	[0.61, 1.47]	.79			
Always	0.90	[0.55, 1.48]	.68			
Number of new female partners, last 3 months						
0	Ref			Ref		
1	1.65	[1.12, 2.44]	.01	1.78	[1.02, 3.10]	.04
≥2	1.35	[0.82, 2.21]	.24	1.43	[0.73, 2.83]	.30
Known HIV positive female sex partner(s), last 3 months						
No	Ref			Ref		
Yes	0.31	[0.07, 1.45]	.14	0.26	[0.05, 1.25]	.09
Do not know HIV status	1.54	[1.07, 2.22]	.02	1.31	[0.86, 1.98]	.21
Alcohol consumption prior to sex, last month						
No	Ref			Ref		
Yes	1.58	[1.11, 2.24]	.01	1.59	[1.09, 2.31]	.02
Age of sexual debut, years						
≥19	Ref					
15–18	1.05	[0.67, 1.64]	.84			
≤14	1.14	[0.63, 2.08]	.66			
History of treatment or symptoms of STI, past 3 months						
No	Ref			Ref		
Yes	1.76	[1.12, 2.78]	.02	1.85	[1.15, 2.99]	.01

*Note.* OR = odds ratio; CI = confidence interval; STI = sexually transmitted infection, self-report of signs/symptoms or being treated for.

a Includes permanent, temporary, self-employment. ^b^Participants who had a female partner 3 months prior to screening but not at baseline. ^c^Includes apprenticeship or studentships. ^d^1–3 times/month or weekly.

## Discussion

We report that nearly half the men in our sample had permissive attitudes toward GBV, and men who reported more HIV risk behavior were more likely to condone violence toward women. We also identified that being young (18–24 years old), having less years of education, and not currently having a sexual partner at baseline were also independently associated with condoning GBV. In a 2017 South African National HIV Survey, key drivers of the HIV epidemic among men aged 15 to 24 years old were reported to be similar to the factors we identified as being associated with GBV (Simbayi et al., 2019). In our study, however, no association between male report of condom use and permissive attitudes toward GBV was identified, although men may have overreported condom use due to social desirability bias. While it is possible that similar socioeconomic and cultural factors support both HIV risk behavior and GBV, we did not collect data on the social context within which these participants lived. Nevertheless, our findings support the integration of GBV and HIV prevention programs (Jewkes et al., 2011; UNICEF, 2017).

Permissive attitudes toward GBV among men in our sample were more prevalent than that reported in the sub-Saharan African (SSA) MICS cross-sectional study conducted between 2005 and 2018. Using the same GBV questionnaire, that survey indicated that 30% of men condoned GBV (UNICEF, 2017), compared with 43% in our study. Several reasons could exist for this difference. Our sample was smaller, less diverse, and not representative of the general population of adult men in SSA. In addition, eligibility for enrolment in our parent study required reporting HIV high risk behavior. To the extent that men with HIV risk behavior were more likely to condone GBV, our sample would be biased toward men who were more permissive of GBV. Other studies performed in Africa have reported that GBV is prevalent in the region (Jewkes et al., 2013; NDoH et al., 2019; World Health Organization, 2004, 2014). In the 2016 Demographic and Health Survey of South Africans, 26% of adult women who were sexually experienced reported being subjected to some form of GBV (NDoH et al., 2019).

Our results suggest that young men may be more likely to condone GBV and could be a key target population for prevention programs (Jewkes et al., 2015). In studies from South Africa (Jewkes et al., 2011), Bangladesh, Sri Lanka, Cambodia, China, Indonesia, and Papua New Guinea (Fulu et al., 2013; Jewkes et al., 2013), the majority of men who indicated ever raping women, first did so during their adolescence (Jewkes et al., 2015). Men justified rape because they believed in “sexual entitlement” with women (Fulu et al., 2013; Jewkes et al., 2011, 2013). Boys exposed to violence are more prone to becoming aggressive and impulsive and are more likely to perpetrate GBV (Jewkes et al., 2015). In a 2011 South African study of 1,737 men aged 18 to 49, perpetration of rape was associated with having a difficult childhood, being raped by other men, having inequitable views on gender relations, being in gangs, and socially condoning patriarchal practices (Jewkes et al., 2011). These studies suggest that intervening early in an adolescent boy’s life may reduce violent behavior toward women. In addition, the malleability of adolescent behavior presents an opportunity to modify attitudes earlier in life and may lead to better results than targeting adults (Jewkes et al., 2015, 2019).

GBV interventions could be implemented by engaging with communities, families, and the individual through health officers or local groups so that all members of the community can better understand how GBV is manifested and its long-ranging effects. Interventions that work with men to address their view of masculinity and identify healthy behaviors, and that mediate dialogue between perpetrators and survivors of GBV, have been reported to improve gender-equity beliefs between men and women (Jewkes et al., 2015). A systematic review of studies of the association between HIV infection and perceptions of masculinity reported that men who valued hegemonic masculinity condoned the demonstration of wealth, sexual prowess and bravado, and prioritized their ability to make money over seeking care and treatment for health (Jacques-Aviñó et al., 2019). In South Africa, these traits have been shown to be important to men (Jewkes et al., 2011) and socially desirable in communities that condone dominance of men over women (Strebel et al., 2006). Intervention strategies should discourage hegemonic masculinity, support gender-equity, and enhance communication and relationship skills (Jewkes et al., 2015).

Our study indicated that men who did not have a current sexual partner at the time of the baseline interview were more likely to report permissive attitudes toward GBV, compared with those who were living with a woman. It is possible that younger men and those living without a woman were less experienced in maintaining a female relationship, and therefore had preconceived ideas about the acceptability of GBV (Jewkes et al., 2011; Strebel et al., 2006). Economic factors may also play a role. Younger men might be more economically vulnerable and more inclined to express their frustration by perpetrating violence against women. More than half of our participants were unemployed and therefore likely to have been financially stressed. Studies of women in sub-Saharan African (Iman’Ishimwe Mukamana et al., 2020; Jewkes et al., 2015, 2019; Nyamhanga & Frumence, 2014) and Asia (Fulu et al., 2013; Jewkes et al., 2013, 2017) have reported that women who were economically dependent on cohabitating male partners were at risk of psychological, physical, and sexual abuse (Iman’Ishimwe Mukamana et al., 2020; Jewkes et al., 2015, 2019; Kiss et al., 2012). Unfortunately, the parent study from which we analyzed data did not ask questions about economic dependency; therefore, we were unable to evaluate whether this also held true in our sample.

Our study had several limitations. The participants from whom we analyzed data may not be representative of the general population of men in this area, and the parent study only enrolled men who were HIV-uninfected. The questionnaire was developed to evaluate GBV toward women and has not been used among MSM; for this reason, we excluded from analysis the small number of men in the parent study who only had sex with other men.

We used the MICS GBV questionnaire because it has been standardized and used globally. However, the instrument is narrowly focused and only asks about attitudes toward GBV and not about whether men had previously perpetrated or anticipate perpetrating violence against women. In addition, the questionnaire only asks about physical violence and does not include questions about sexual, psychological, or emotional violence, economic abuse, or violence toward a non-spousal partner.

The parent study from which we analyzed data was not primarily designed to evaluate factors associated with GBV; therefore, our analysis was constrained by the measurements in the parent study. For this reason, we could not explore underlying factors that might be associated with GBV, such as traumatic childhood experiences, violence, or stress. Although it is possible that adoption of or belief in traditional attitudes toward masculinity may support GBV, we did not have any data that allowed us to identify or quantify participants’ views on being “masculine.” Because the questionnaire focused on an individual’s beliefs, we could not determine how characteristics of family, peers, and society may have been associated with attitudes toward GBV.

Nevertheless, this study reports several important findings. The components of the GBV questionnaire showed high internal consistency among men in our sample. Although this scale has not been widely used in South Africa (Cappa, 2014; Lansford et al., 2014), our study supports its use for other studies or interventions among men in this region. In addition, our data add to the limited literature on identifying men who may be ideal candidates for integrated HIV and GBV programs.

## Conclusion

Screening men about their attitudes toward GBV could aid in identifying those who are more prone to perpetrating violence toward women (ANA Reporter, 2020; Glover et al., 2020; Joska et al., 2020; Ndaba, 2020; UNAIDS, 2020a). Our results support other studies that have reported a relationship between GBV and risky HIV sexual behavior. Targeting adolescent boys and young men may be particularly beneficial in preventing both HIV and GBV. Combined interventions may be especially needed during the current COVID-19 pandemic, which has resulted in an increase in domestic violence in South Africa.

## Supplemental Material

sj-doc-1-jmh-10.1177_15579883221106331 – Supplemental material for Attitudes Toward Gender-Based Violence Among Sexually Active Adult Men at High Risk for HIV in Rustenburg, South Africa Click here for additional data file. Supplemental material, sj-doc-1-jmh-10.1177_15579883221106331 for Attitudes Toward Gender-Based Violence Among Sexually Active Adult Men at High Risk for HIV in Rustenburg, South Africa by Heeran Makkan, Pholo Maenetje, Candice M. Chetty-Makkan, Evans Muchiri, Mary H. Latka, Vinodh A. Edward, Matt A. Price, Gloria Omosa-Manyonyi and Christina Lindan in American Journal of Men’s Health

sj-doc-2-jmh-10.1177_15579883221106331 – Supplemental material for Attitudes Toward Gender-Based Violence Among Sexually Active Adult Men at High Risk for HIV in Rustenburg, South Africa Click here for additional data file. Supplemental material, sj-doc-2-jmh-10.1177_15579883221106331 for Attitudes Toward Gender-Based Violence Among Sexually Active Adult Men at High Risk for HIV in Rustenburg, South Africa by Heeran Makkan, Pholo Maenetje, Candice M. Chetty-Makkan, Evans Muchiri, Mary H. Latka, Vinodh A. Edward, Matt A. Price, Gloria Omosa-Manyonyi and Christina Lindan in American Journal of Men’s Health
