# Supplementary material for: Attitudes Toward Gender-Based Violence Among Sexually Active Adult Men at High Risk for HIV in Rustenburg, South Africa
Source: Am J Mens Health. 2022 Jun 24;16(3):15579883221106331. doi: 10.1177/15579883221106331 (PMC9234859; doi:10.1177/15579883221106331)
Supplement: sj-doc-1-jmh-10.1177_15579883221106331 – Supplemental material for Attitudes Toward Gender-Based Violence Among Sexually Active Adult Men at High Risk for HIV in Rustenburg, South Africa [file sj-doc-1-jmh-10.1177_15579883221106331.doc]

**Start time: ________:______**

**Aurum Screening Questionnaire**

- **Thank you for agreeing to take part in our research.**
- **We are interested in knowing about people’s health. Some of these questions are sensitive, but it is very important that we know the truth about what is going on in this community. There are no right or wrong answers. Please be honest when answering these questions.**
- **You do not have to answer any questions if you don’t want to – what is most important is that you feel comfortable answering honestly.**
- **Your answers to these questions will tell us about the community which will help us for future research projects.**
- **All of your answers will be kept confidential. Confidential means we will not tell your answers to anyone outside the research group.**

| Research Assistant] | |  | | | **CODE** |
| --- | --- | --- | --- | --- | --- |
| D1 | **How did you first hear about the study? Please pick one of the options from this card.** | 1. In a clinic 2. Approached in my neighborhood 3. Through mobile VCT 4. Through a community meeting or out in community 5. Heard about it from a friends / family 6. From my partner 7. From school 8. Walk in 9. Tavern/shebeen/ place where alcohol is consumed 10. Other (specify below) | | |  |
| D2 | If code 10 above,,write where they heard about Aurum from here 🡪 |  | | | |
| D3 | **How old are you?** | Code in years🡪 | | |  |
| D4 | **What is your date of birth?** | Write in date | ___ ___ / ___ ___ ___ / ___ ___ ___ ___  DAY / M O N / Y Y Y Y | | |
| D5 | **In which area of Rustenburg do you live?** Write area here🡪 |  | | | |
| D6 | **What is your gender? That is, are you male, female, or transgender or transsexual?** | 0= Male  1= Female  2= Transgender/ transsexual | | |  |
| D7 | **How would you describe your employment situation? Please pick from this card.** | 0= Unemployed looking for work   1. Unemployed not looking for work 2. Unable to work - receive social grant 3. Student/pupil/learner 4. Self-employed – part time less than 40 hours per week 5. Self-employed – full time 40 hours or more per week 6. Employed, less than 40 hours per week 7. Employed, full time (40 hours or more) 8. Other | | |  |
| D7o | If code 8 above, write employment situation🡪 |  | | | |
| D8 | **How would you describe your family’s financial situation?** | 1. Not enough money for basic things like food, clothes 2. Have money for food and clothes but short on many other things 3. We have the basics but not enough money for expensive things 4. Have money to save or buy expensive things 5. Other | | |  |
| D8o | If code 5 above, write family situation 🡪 |  | | | |
| D9 | **Compared with other families would you say that your family is financially better off, about the same, or poorer than others?** | | | 0= Poorer than others  1= About the same as others  2= Better off than others |  |
| D10 | **Compared with your peers would you say that you are financially better off, about the same, or poorer than your peers?** | | | 0= Poorer than others  1= About the same as others  2= Better off than others |  |

|  |  |  | | | | | | | **CODE** |
| --- | --- | --- | --- | --- | --- | --- | --- | --- | --- |
| D11  DEM2 8 | **What has been your primary source of income in the last 12 months? (code up to 2 choices: first choice here, second choice in next question)** | | | | 1. Family 2. Formal employment 3. Casual labour 4. Spouse, boyfriend, girlfriend 5. Self-employment 6. None 7. Other | | | |  |
| D12  DEM2 8o | If code 7 above Write primary source of income here🡪 |  | | | | | | | |
| D13  DEM2 8 | **Any other main source of income?** | | | | 1. Family 2. Formal employment 3. Casual labour 4. Spouse, boyfriend, girlfriend 5. Self-employment 6. None 7. Other | | | |  |
| D14  DEM28o | If code 7 above Write primary source of income here🡪 |  | | | | | | | |
| D15  DEM2 9 | **Are you currently caring (financially) for any dependents?** | | | | | 0= No  1=Yes | |  | |
| D16 | **How would you describe your relationship status?**  **[Pick from card]** | 1. Married (civil magistrate /traditional /religious) 2. Not married but living together 3. Single but have a steady partner 4. Single but have steady and casual partner(s) 5. Single but have casual partner(s) 6. Single with no partners 7. Other | | | | | |  | |
| D16o | **If code 7 above** | Write relationship status here🡪 | | | | |  | | |
| D17 | **Does your partner live with you?** | | 0= No  1=Yes | | | | |  | |
| D18 | **[If has only one partner of any kind]**  **How long have you been with this partner?** | | | Code in months for all time together. Take out time not together.  Code 0 if one time partner 🡪 | | | |  | |
| D19 | **[If any partners]**  **In the last month, how often have you seen your partner(s)?** | | 1. Less than once a month 2. About once a month 3. About two to three times a month 4. Once a week 5. More than once a week | | | | |  | |
| D20 | **Please tell me which of these statements best describes your sexual orientation?** | | 1. Heterosexual 2. Homosexual 3. Bisexual 4. Heterosexual but sometimes have sex with the same sex 5. Homosexual but sometimes have sex with the opposite sex 6. No orientation, I sleep with whomever I like | | | | |  | |

**Now some questions about you**

|  | | | | | | | **CODE** |
| --- | --- | --- | --- | --- | --- | --- | --- |
| DEM11 | **What is your race?** | 1= Black  2= White  3= Asian  4= Other (specify below) | | | | |  |
| DEM11o | Write in other here🡪 |  | | | | | |
| DEM12 | **What is your tribal or ethnic group?** | | Write in name here 🡪 | |  | | |
| DEM 13 | **What is your country of birth?** | | Write in name here 🡪 | |  | | |
| **How many total years of school have you completed in each category?** | | | | | | | |
| DEM14a | **a. Primary** | | | Code number of years 🡪 | | |  |
| DEM14b | **b. Secondary** | | | Code number of years 🡪 | | |  |
| DEM 14c | **c. Post-secondary** | | | Code number of years 🡪 | | |  |
| DEM14d | **d. Completed years of other school/apprenticeship** | | | Code number of years 🡪 | | |  |
| DEM14e | **If has other school/apprenticeship, specify type here🡪** | | |  | | | |
| DEM15 | **What is your religion?** | | | 1= Catholic  2= Protestant  3= Other Christian  4= Muslim  5= None  6= Other | | |  |
| DEM15o | If above is other specify other religion here🡪 | | | | |  | |
| DEM16 | **How many years have you lived in this area** | Code number of years; code= 00 if less than 1 year 🡪 | | | | |  |
| DEM17 | **What is your current marital status?** | 1= Single  2= Divorced/separated  3= Married, monogamous  4= Married, polygamous  4= Widowed | | | | |  |

| **Now some questions about things you may or may not own. These questions refer to things that you personally own (not about your household).** | | |
| --- | --- | --- |
| **Do you personally own a…** | | **USE THESE CODES**  **0= No**  **1= Yes** |
| PP1 | **...working wrist watch?** |  |
| PP2 | **…jewelry?**  (ring, necklace, chains made of gold, silver platinum etc.) |  |
| PP3 | **…working cell phone?** (any type) |  |
| PP4 | **…working cell phone that has a camera?** |  |
| PP5 | **….working cell phone that connects to internet?** (Facebook, Twitter, MXIT) |  |
| PP6 | **…working iPod / personal music device / MP3 player?** |  |
| PP7 | **...working computer?**  (laptop/desktop; any kind of working computer=yes) |  |
| PP8 | **…working iPad** |  |
| PP9 | **…working private car** |  |
| PP10 | **…working taxi or similar vehicle using to commute passengers** |  |
| PP11 | **…working motor bike /scooter?** |  |
| PP12 | **… a house – either alone or jointly with someone else?** |  |
| PP13 | **... any land – either alone or jointly with someone else?** |  |
| PP14 | **… a cheque or saving account at a bank?** |  |
| PP15 | **... a bank credit card?** |  |
| PP16 | **…a loan from a cash loan store?** |  |
| PP17 | **… any kind of store credit card?** |  |

|  |  |  | **CODE** |
| --- | --- | --- | --- |
| PP18 | **How often do you save money?** | **0**=Not saving  1= Save once a year (includes 13^th^ cheque)  2= Every six months or so  3= Every few months  4= Save every month |  |

| **Now let’s talk about things you may or may not have in your household. When I ask about household I am talking about the place where you mainly stay right now– not about your home village or where you come from. For these questions, I am talking about things that are in working condition.** | | |
| --- | --- | --- |
| **Does your household have a working…** | | **USE THESE CODES**  **0= No**  **1= Yes** |
| HH1 | **… TV?** (any kind) |  |
| HH2 | **… plasma or flat screen TV?** |  |
| HH3 | **…DSTV, MNET or TopTV, or other satellite television connection?** |  |
| HH4 | **…an entertainment system / sound system?** |  |
| HH5 | **…working fridge?** |  |
| HH6 | **…working landline telephone?** |  |
| HH7 | **…working electricity?** |  |
| HH8 | **…working water tap in house?** |  |
| HH9 | **…working water tap in yard?** |  |

| **Now I am going to read you a list about supporting others. Please tell me how often you have done any of these things in the last 3 months.** | | |
| --- | --- | --- |
|  | **In the last 3 months, how often did you…** | **READ CODES OUT LOUD TO PARTICIPANT**  0=Never  1= A few times  2= Monthly or more |
| FS1 | **…buy your family food for the house?** |  |
| FS2 | **…give food, money or things to support your family household?** |  |
| FS3 | **…give food, money or things to support other family members (outside your immediate household)?** |  |
| FS4 | **…give food, money or things to support friends?** |  |
| FS5 | **…give food, money or things to help support a lover (who is not part of your household as per above)?** |  |
| FS6 | **…give food, money or things to help support a someone you want as a lover?** |  |

**ASK OFALL—REGARDLESS OF ANY ANSWERS GIVEN ABOVE ABOUT EMPLOYEMENT**

| Now I’m going to ask you some questions about your working situation. You do not have to answer any question that you don’t want to. There are no right or wrong answers to these questions. Please be honest when you answer. Like the census, we are asking because we want to better understand this community. | | | | |
| --- | --- | --- | --- | --- |
| E1 | **What is your job? That is, what kind of work do you do?** | Write in job here and be as descriptive as possible. If no job write not working | | |
| E2 | **How many hours, including overtime, did you work during the last 7 days?** | Code number of hours worked (if did not work, code =0)🡪 | | **CODE** |
| E3 | **How many hours per week, including overtime, do you usually work?** | Code number of hours worked (if did not work, code =0)🡪 | |  |
| E4 | **Do you want to work longer hours than you currently are working?** | 0= No  1= Yes | |  |
| E5 | **Do you usually work throughout the year, or do you work seasonally, or only once in a while?** | 0= Work once in a while  1= Works seasonally  2= Works throughout the year | |  |
| E6 | **How are you paid for work done, or are you not paid at all?** | 0= Not paid  1= Paid in kind only (get things/services not money)  2= Paid in cash and kind  3= Paid in cash only | |  |
| E7 | **How much job security do you feel you have?** | 0= No job security  1= Some job security  2= Pretty good job security  3= Very secure job | |  |
| E8 | **Please tell me the letter that matches your total monthly pay for your main job (include overtime, allowances, bonuses)?**  **[hand out card,**  **read options before asking question]** | In Rands,   1. 0 – 2,000 2. 2,001 – 5,000 3. 5,001 – 10,000 4. 10,001 – 15,000 5. 15,001 – 20,000 6. More than 20,000 | **Code letter here** | |

| **Now let’s talk about your social life. Please tell me how often you have done these thing in the last 3 months.**  **[hand out card, read options before asking questions]** | | |
| --- | --- | --- |
|  | **In the last 3 month how often did you…** | **READ CODES OUTLOUD TO PARTICIPANT**  0=Never  1=Rarely (have done, but not regularly)  2= Sometimes (do this from time to time)  3= Often (I do this weekly)  4= Regularly (do this more than 1 time per week ) |
| SL1 | **…buy a prepared meal at a tuckshop, take away or restaurant for yourself?** |  |
| SL2 | **…buy a prepared meal at a tuckshop, take away or restaurant for family members?** |  |
| SL3 | **…buy a prepared meal at a tuckshop, take away or restaurant for your friends?** |  |
| SL4 | **…buy a prepared meal at a tuckshop, take away or restaurant for your lovers?** |  |
| SL5 | **…buy a prepared meal at a tuckshop, take away or restaurant for someone you want as a lover?** |  |
| SL6 | **...give gifts/things other than food/drink to a lover?** |  |
| SL7 | **...give giftst/things other than food/drink to someone you want as a lover?** |  |
| SL8 | **…spend time at a tavern/ shebeen?** |  |
| SL9 | **…spend time hanging out at shopping centers/ hair salons/ or tuckshops?** |  |
| SL10 | **… spend time card playing or gambling?** |  |
| SL11 | **…spend time at informal car washes or picnic spots?** |  |
| SL12 | **…buy yourself a drink at a tavern/ shebeen/ place to drink alcohol?** |  |
| SL13 | **…buy your friends a drink at a tavern/ shebeen/ place to drink alcohol?** |  |
| SL14 | **…buy a someone you want as a lover a drink at a tavern / shebeen/ place to drink alcohol?** |  |

|  | | | | **CODE** |
| --- | --- | --- | --- | --- |
| RIS11 | **During the last month, on average, how often have you had a drink containing alcohol?** | 0= None  1= 1-3 times per month  2= Weekly  3= Daily | |  |
| RIS12 | **During the last month, how often were you drunk/drinking alcohol before sex?** | 0= Never  1= Sometimes (less than half)  2= Frequently (more than half)  3= Always | |  |
| **\**  **Some people have tried a range of different types of drugs. In the last month, which of the following, if any, have you used?** | | | | |
| RIS13a | **Khat/ Miraa** | | 0= No  1= Yes  88= don’t know |  |
| RIS13b | **Injecting drugs with a needle** | | 0= No  1= Yes  88= don’t know |  |
| RIS13c | **Marijuana** | | 0= No  1= Yes  88= don’t know |  |
| RIS13d | **Other** | | 0= No  1= Yes  88= don’t know |  |
| RIS13do | Specify other drug use here 🡪 | |  | |

**[For women only ask:] Now some questions about your family and reproductive history. (For males code=97)**

| Now some questions about family life… | | | | **CODE** |
| --- | --- | --- | --- | --- |
| PG1 | **Have you ever been pregnant?** | 0 = No  1 = Yes | |  |
| PG2 | **If yes, how old were you when you fell pregnant the first time?** | Code age as 97 if never pregnant. | |  |
| PG3 | **Are you pregnant right now?** | 0 = No  1 = Yes  2 = Not sure | |  |
| PG4  DEM210a | **How many total past pregnancies have you had?** | WRITE number 🡪  USE IAVI rules for NA. | |  |
| PG5 | **How many of these pregnancies were planned?** | WRITE number here**🡪**  97 if never pregnant. | |  |
| PG6  DEM210b | **How many live births have you had?** | WRITE number here**🡪**  USE IAVI rules for NA. | |  |
| PG7 | **When was the last time you gave birth to a living child?** | WRITE Mo & Yr here**🡪** | **_____ /_____**  **MON / YYYY** | |
| PG8  DEM210c | **How many of your children died between the ages of 0 and 5 years of age?** | WRITE number here**🡪**  USE IAVI rules for NA | |  |
| PG9 | **How many biological children do you have? That means your own children.** | WRITE number here**🡪** | |  |
| PG10 | **Are you satisfied with the number of children that you currently have?** | 1 = Very unsatisfied  2 = Slightly unsatisfied  3= No feelings / neutral  4 = Satisfied  5 = Very satisfied | |  |
| PG11 | **How many children in total would you like to have?** | WRITE number here**🡪** | |  |
| PG12 | **How many different biological fathers are there for your children?** | WRITE number here**🡪**  97 if never pregnant. | |  |
| PG13 | **In the last 3 months, have you or your partner wanted to fall pregnant?**  **[Read options if needed]** | 1 = I wanted to, but my partner didn’t  2 = I did not want to, but my partner did  3 = Both wanted to  4 = Both did not want to | |  |
| PG14 | **Do you or your partner want to fall pregnant in the next year?**  **[Read out options]** | 1 = I want to, but my partner doesn’t  2 = I do not want to, but my partner does  3 = We both want to  4 = We both do not want to | |  |
| PG15 | **What is the best age for a female to fall pregnant for the first time?** | WRITE age here**🡪** | |  |
| PG16 | **What is the best age for a man to father a child?** | WRITE age here**🡪** | |  |
| PG17 | **How old were you when you first had sex (vaginal or anal)?** | WRITE age here**🡪** | |  |

**[For men only ask:] Now some questions about your family and reproductive history. (For females code=97)**

|  | | | | **CODE** |
| --- | --- | --- | --- | --- |
| PG1 | **Have you ever gotten a woman/ girl pregnant?** | 0 = No  1 = Yes  2= Not sure | |  |
| PG2 | **If yes, how old were you when you got someone pregnant the first time?** | Code 99 if never created pregnancy. | |  |
| PG3 | **Are any of your partners pregnant right now?** | 0 = No  1 = Yes  2 = Not sure | |  |
| PG4 | **How many pregnancies have you been responsible for (no matter the outcome)?** | WRITE number here**🡪**  97 if never created a pregnancy. | |  |
| PG5 | **How many of these pregnancies were planned?** | WRITE number here**🡪**  97 if never created a pregnancy. | |  |
| PG6 | **How many live births have you been the biological father to?** | WRITE number here**🡪**  97 if never created a pregnancy. | |  |
| PG7 | **When was the last time you fathered a living child?** | WRITE Mo&Yr here**🡪** | **_____ /_____**  **MON / YYYY** | |
| PG8 | **How many of your children died between the ages of 0 and 5 years of age?** | WRITE number here**🡪**  97 if never fathered a child | |  |
| PG9 | **How many biological children do you have? That means your own children.** | WRITE number here**🡪** | |  |
| PG10 | **Are you satisfied with the number of children that you currently have?** | 1 = Very unsatisfied  2 = Slightly unsatisfied  3= No feelings / neutral  4 = Satisfied  5 = Very satisfied | |  |
| PG11 | **What is your ideal number of children?** | WRITE number here**🡪** | |  |
| PG12 | **How many different biological mothers are there for your children?** | WRITE number here**🡪**  97 if never fathered a child | |  |
| PG13 | **In the last 3 months, have you or your partner wanted to fall pregnant? [Read options if needed]** | 1 = I wanted to, but my partner didn’t  2 = I did not want to, but my partner did  3 = Both wanted to  4 = Both did not want to | |  |
| PG14 | **Do you or your partner want to fall pregnant in the next year? [Read out options]** | 1 = I want to, but my partner doesn’t  2 = I do not want to, but my partner does  3 = We both want to  4 = We both do not want to. | |  |
| PG15 | **What is the best age for a female to fall pregnant for the first time?** | WRITE age here**🡪** | |  |
| PG16 | **What is the best age for a male to father a child?** | WRITE age here**🡪** | |  |
| PG17 | **How old were you when you first had sex (vaginal or anal)?** | WRITE age here**🡪** | |  |

**Now I’m going to ask you about your beliefs. People have many different beliefs. First, I would like to hear your beliefs about condoms. I am going to read you a statement, please tell me how much you agree or disagree with this statement using a number from this card. Remember there are no right or wrong answers. Please tell me your opinion.**

**[Hand out card; explain how to use it]**

|  |  | **Strongly disagree** | **Disagree** | **Agree** | **Strongly**  **Agree** |
| --- | --- | --- | --- | --- | --- |
| CU1 | **I am able to convince my partner to use condoms even if s/he or she does not want to.** | **1** | **2** | **3** | **4** |
| CU2 | **I would remember to use a condom even after I have been drinking.** | **1** | **2** | **3** | **4** |
| CU3 | **I could stop to put a condom on myself or my partner even if we are both very sexually aroused.** | **1** | **2** | **3** | **4** |
| CU4 | **I can refuse sex when there is no condom available.** | **1** | **2** | **3** | **4** |
| CU5 | **If I were to suggest using condoms with a partner that I hadn’t been using condoms with, I would feel afraid that s/he would reject me.** | **1** | **2** | **3** | **4** |
| CU6 | **If I suggest using condoms with a new partner then s/he would think I have a sexually transmitted disease.** | **1** | **2** | **3** | **4** |
| CU7 | **If I suggest using condoms with a new partner then s/he would think I thought they had a sexually transmitted disease.** | **1** | **2** | **3** | **4** |

**Now some questions about your beliefs related to pregnancy. Please tell me how much you agree or disagree with the following statements. [Repeat instructions and reminders]**

|  | **[Provide response card]** | **Strongly disagree** | **Disagree** | **Agree** | **Strongly**  **Agree** |
| --- | --- | --- | --- | --- | --- |
| PB1 | **A girl must fall pregnant to show that she is fertile.** | 1 | 2 | 3 | 4 |
| PB2 | **A man will only want to marry a girl if she has been made pregnant by him before.** | 1 | 2 | 3 | 4 |
| PB3 | **A girl is not a woman unless she has had a child.** | 1 | 2 | 3 | 4 |
| PB4 | **A boy is not a man until he has had a child with a woman.** | 1 | 2 | 3 | 4 |
| PB5 | **It is a family shame if a girl falls pregnant before being married.** | 1 | 2 | 3 | 4 |
| PB6 | **A man will only want to marry a girl if she has borne his child/children.** | 1 | 2 | 3 | 4 |
| PB7 | **Parents get a smaller lobola if their daughter was pregnant and had a child.** | 1 | 2 | 3 | 4 |
| PB8 | **Females fall pregnant so that they can obtain a government child grant.** | 1 | 2 | 3 | 4 |
| PB9 | **A woman believes that she must fall pregnant to show that she is a real woman.** | 1 | 2 | 3 | 4 |
| PB10 | **Friends influence each other to fall pregnant.** | 1 | 2 | 3 | 4 |
| PB11 | **Pregnancy is a sign of femininity.** | 1 | 2 | 3 | 4 |
| PB12 | **Men believe that a woman must fall pregnant to show that she is a real woman.** | 1 | 2 | 3 | 4 |

**Now some questions about your beliefs about relationships between men and women.**

|  | **[Provide response card]** | **Strongly**  **Disagree** | **Disagree** | **Agree** | **Strongly Agree** |
| --- | --- | --- | --- | --- | --- |
| R1 | Men have many lovers because it is in their nature to do so | 1 | 2 | 3 | 4 |
| R2 | Men have lovers to get energy to satisfy their primary partners | 1 | 2 | 3 | 4 |
| R3 | Women these days say that they need to have more than one sex partner | 1 | 2 | 3 | 4 |
| R4 | Men feel ashamed of their wives and want young lovers to take around to their friends | 1 | 2 | 3 | 4 |
| R5 | If men do not have lovers their friends laugh at them | 1 | 2 | 3 | 4 |
| R6 | Women who are financially independent do not want to commit themselves to one relationship | 1 | 2 | 3 | 4 |
| R7 | The families of young people who work do not want them to get married because they are afraid to lose their income | 1 | 2 | 3 | 4 |
| R8 | Men often force women in subtle ways to have sex with them even if they do not want to | 1 | 2 | 3 | 4 |
| **A husband is justified in hitting or beating his wife if she…** | |  |  |  |  |
| PWR1 | …goes out without telling him. | 1 | 2 | 3 | 4 |
| PWR2 | …neglects the children. | 1 | 2 | 3 | 4 |
| PWR3 | …argues with him. | 1 | 2 | 3 | 4 |
| PWR4 | … refuses to have sex with him. | 1 | 2 | 3 | 4 |
| PWR5 | …burns the food. | 1 | 2 | 3 | 4 |

**Finally, some questions about ways to prevent HIV.**

|  | | **CODE**   1. No 2. Yes   2= Not sure |
| --- | --- | --- |
| K1 | Is there currently a vaccine that stops people from getting infected with HIV? |  |
| K2 | Is there currently a vaccine that slows down disease after people are infected with HIV? |  |
| K3 | Is there currently a gel that women can put in their vaginas and stop them from getting HIV during sex? |  |
| K4 | If man has had his penis circumcised by a doctor is he less likely to get infected with HIV? |  |

**This is the end of the questionnaire. Thank you for taking the time to answer these questions.**

| TIMEEND | **Time Interview Ended** | HH:MM using 24 hour clock | **___ ___ : ___ ___** |
| --- | --- | --- | --- |

[Refer as needed. Correct knowledge as needed using text below]:

- I want to make sure that you know that there is currently no vaccine to stop or slow down HIV.
- There is currently no gel product that women can put in their vaginas to stop them from getting HIV during sex.
- Vaccines and gels are being tested in South Africa to find out if they work. But right now we do not have a vaccine or a gel that works.
- Men who have had their penises circumcised by a doctor are at lower risk of getting HIV. I can give you the contact details of a doctor who does circumcision.

INFORMATION TO BE COMPLETED BY INTERVIEWER AFTER INTERVIEW:

|  |  |  | | CODE |
| --- | --- | --- | --- | --- |
| I1 | **Interviewer code for interviewer** |  | |  |
| I2 | **In what language was the interview administered?** | 0=English  1=Tswana  2=Afrikaans  3=Mix of English & Tswana  4=Xhosa  5=Zulu  6=Other | |  |
| I2o |  | If other specify🡪 |  | |

**INFORMATION TO BE COMPLETED BY STUDY COORDINATOR OR DESIGNEE:**

|  |  | **CODE** |
| --- | --- | --- |
| **Is the participant eligible for IAVI Protocol B?** | 0= Ineligible   1. Eligible   97= Not applicable |  |
