# Supplementary material for: Attitudes Toward Gender-Based Violence Among Sexually Active Adult Men at High Risk for HIV in Rustenburg, South Africa
Source: Am J Mens Health. 2022 Jun 24;16(3):15579883221106331. doi: 10.1177/15579883221106331 (PMC9234859; doi:10.1177/15579883221106331)
Supplement: sj-doc-2-jmh-10.1177_15579883221106331 – Supplemental material for Attitudes Toward Gender-Based Violence Among Sexually Active Adult Men at High Risk for HIV in Rustenburg, South Africa [file sj-doc-2-jmh-10.1177_15579883221106331.doc]

**Start time: ________:______**

**hh mm**

**AURUM CLINICAL ASQ**

| **In the last 3 months…** | |  | **CODE** |
| --- | --- | --- | --- |
| STI1 | **…has a doctor or a nurse diagnosed you for any STI?** | 0= No  1= Yes |  |
| STI2 | **…have you been treated for an STI?** | 0= No  1= Yes |  |
| STI3  RIS14 | **…have you had any smelly or abnormal vaginal or penile discharge?** | 0= No  1= Yes |  |
| STI4 | **…have you had any painful urination?** | 0= No  1= Yes |  |
| STI5 | **…have you had pain during intercourse?** | 0= No  1= Yes |  |
| STI6  RIS15 | **…have you had any sores or ulcers on your vagina or penis?** | 0= No  1= Yes |  |
| STI7 | **IF FEMALE…have you had any lower abdominal pain?**  **Code=97 if male** | 0= No  1= Yes |  |
| STI8 | **IF FEMALE: …have you had vaginal itching or burning?**  **Code=97 if male** | 0= No  1= Yes |  |

**ASK OF MEN ONLY (if female code = 97)**

| **Now some questions about circumcision of your penis—a procedure where your foreskin is removed.** | | | **CODE** |
| --- | --- | --- | --- |
| Cir1 | **Have you ever had your penis circumcised by a doctor or nurse?** | 0= No  1= Yes |  |
| Cir2 | **[If yes, circumcised by doctor/nurse]**  **How old were you when you had circumcision by a doctor or nurse?** | Code age  in years |  |
| Cir3 | **[If no, not medically circumcised]**  **Would you like a referral to a doctor who can do circumcision?** | 0= No  1= Yes |  |
| CIR4 | **Have you ever had your penis circumcised in a cultural practice?** | 0=No  1=Yes |  |
| CIR5 | **[If circumcised in cultural practice]**  **How old were you when you had cultural circumcision?** | Code age  in years |  |

**Now I’m going to ask you about things you or your partner may have used in the last 3 months. If you’ve had one partner in the last 3 months, please think of that person when answering these questions. If you have had more than one partner in the last three months, please think of the partner you had sex with the** most.

| **Now I’m going to read you a list. Please tell me if you or your partner have used any of the following methods in the last 3 months…** | | **CODES**  0=No  1=Yes  88=Don’t Know  97= not applicable, no sex |
| --- | --- | --- |
| C1 | **hormonal injection (depo)** |  |
| C2 | **hormonal contraceptive pills (the pill)** |  |
| C3 | **had an (induced) abortion** |  |
|  | **Now please tell me how often you or your partner have used any of the following methods in the last 3 months…** | **CODES**  0=Never  1=Sometimes  2= half the time  3= Most of the time  4=Almost all the time  5= Absolutely every single time  97= not applicable, no sex |
| C4 | **male condoms** |  |
| C5 | **female condoms** |  |
| C6 | **Pulled out the penis before ejaculation, or spermed outside the vagina** |  |

| **Have you or your partner ever had a …** | | **CODES**  0=No  1=Yes |
| --- | --- | --- |
| C7 | **tubal ligation (woman sterilized or tubes tied)** |  |
| C8 | **hysterectomy (operation to take out woman’s womb or uterus )** |  |
| C9 | **vasectomy (man sterilized)** |  |

| **Now I’m going to ask you about how often you had sex in the last week, that is, the last 7 days.** | | | **CODE** |
| --- | --- | --- | --- |
| LW1 | **How many times have you had vaginal sex in the last 7 days, including today?** | Code number of sex acts 🡪 |  |
| LW2 | **Is this number of vaginal sex acts in one week typical (your normal amount)?** | 1. No 2. Yes |  |
| LW3 | **How many times have you had anal sex in the last week?** | Code number of sex acts 🡪 |  |
| LW4 | **Is this number of anal sex acts in one week typical (your normal amount)?** | 1. No 2. Yes |  |

| **ASK THESE OF PEOPLE WHO SAID THEY HAVE PULLED OUT THE PENIS IN LAST 3 MOS** | | | | | | **CODE** |
| --- | --- | --- | --- | --- | --- | --- |
| CIY1 | **Whose idea was it to pull out the penis?** | | | | 1. My idea 2. Partner’s idea 3. Both 4. Neither – just happened 5. Other |  |
| CIY2 | **If code other above** **Write reason for pulling out here 🡪** |  | | | | |
| CIY3 | **Did you and your partner communicate about pulling out the penis before or during the sex act?** | | | | 1. No 2. Yes |  |
| CIY4 | **IF YES – Tell me how this topic came up, and what was discussed 🡪** |  | | | | |
| CIY5 | **Why did you or your partner pull out the penis?**  **[DO NOT READ ANSWERS OUT ALOUD – LET PARTICPANT ANSWER FIRST. USE OTHER IF NECESSARY]** | | | 1. To prevent HIV 2. To prevent STIs 3. To prevent pregnancy 4. To prevent both preg & disease 5. Don’t know why–just happened 6. For cultural reasons – specify below 7. Other | |  |
| CIY6 | **If coded as ‘cultural’ or other above** **write reason for pulling out here 🡪** |  | | | | |
| CIY7 | **How long have you been with this partner (that you have pulled out the penis with?)** | | | | Code in months for all total time together (take out times not together).  Code 0 if one time partner 🡪 |  |
|  | **The last time you pulled out the penis, please tell me anything else you were using at the same time…** | | | |  | **CODES**  0=No  1=Yes |
| CIY8 | **…hormonal injection (depo)** | | | | |  |
| CIY9 | **…hormonal contraceptive pills (the pill)** | | | | |  |
| CIY10 | **…male condoms** | | | | |  |
| CIY11 | **…female condoms** | | | | |  |
| CIY12 | **…traditional** | | | | |  |
| CIY13 | **If traditional specify 🡪** | |  | | | |
| CIY14 | **…other? Specify🡪** | |  | | | |
| CIY15 | **Was this typical for what you normally use when pulling out the penis?** | | | |  |  |

| **ASK THESE OF PEOPLE WHO SAID THEY HAVE NOT PULLED OUT THE PENIS IN LAST 3 MOS** | | | | | | | | **CODE** |
| --- | --- | --- | --- | --- | --- | --- | --- | --- |
| CIN1 | **Why not?**  **Write reason for not using pulling out penis here 🡪** | | |  | | | | |
| CIN2 | **Have you ever used pulling out the penis?** | | | | | | 1. No 2. Yes |  |
| CIN3 | **[IF YES TO EVER USE]**  **Tell me how this topic came up, and what was discussed** | |  | | | | | |
| CIN4 | **[IF YES TO EVER USE]**  **Why did you or your partner pull out the penis?**  **[DO NOT READ ANSWERS OUT LOUD – LET PARTICPANT ANSWER FIRST]** | | | | 1. To prevent HIV 2. To prevent STIs 3. To prevent pregnancy 4. To prevent both preg & disease 5. Don’t know why–just happened 6. Other | | |  |
| CIN5 | **[If code other above** **write reason for pulling out here] 🡪** |  | | | | | | |
| CIN6 | **How long were you with the partner (that you have pulled out the penis with)?** | | | | | Code in months for all total time together (take out times not together).  Code 0 if one time partner. 🡪 | |  |
| CIN7 | **How old were you the last time you used pulling out the penis?** | | | | | Code age here 🡪 | |  |

**ASK THIS PAGE TO ALL PARTICIPANTS – NO MATTER WHAT THEIR PRACTICE IS – THERE ARE NO RIGHT OR WRONG ANSWERS HERE – THESE MEASURE PARTICIPANT BELIEFS –NOT PRACTICES**

**Now I would like to hear your beliefs about pulling out the penis before ejaculation, or sperming outside. [Repeat instructions and reminders]**

|  | **[Provide response card]** | **Strongly disagree** | **Disagree** | **Agree** | **Strongly**  **Agree** |
| --- | --- | --- | --- | --- | --- |
| WDMP | There have been times that I have wanted to pull out the penis before ejaculation with my steady/main partner(s) | **1** | **2** | **3** | **4** |
| WDCP | There have been times that I have wanted to pull out the penis before ejaculation with my casual partner(s) | **1** | **2** | **3** | **4** |
| WD1 | I am able to convince my partner to pull out the penis (sperm outside) even if he or she does not want to. | **1** | **2** | **3** | **4** |
| WD2 | I would remember to pull out the penis before/even after I have been drinking alcohol. | **1** | **2** | **3** | **4** |
| WD3 | I could make sure to pull out the penis (sperm outside) even if we are both very sexually aroused. | **1** | **2** | **3** | **4** |
| WD4 | I can refuse sex if there is no chance of pulling out the penis. | **1** | **2** | **3** | **4** |
| WD5 | If I were to suggest pulling out the penis (sperming outside) with a partner I had never done this with before, I would feel afraid that my partner would reject me. | **1** | **2** | **3** | **4** |
| WD6 | If I suggest pulling out the penis my partner would think I have a sexually transmitted disease. | **1** | **2** | **3** | **4** |
| WD7 | If I suggest pulling out the penis a new partner then he or she would think I thought they had a sexually transmitted disease. | **1** | **2** | **3** | **4** |
| WD8 | If I suggest pulling out the penis my partner would think I don’t want to fall / get her pregnant. | **1** | **2** | **3** | **4** |
| WD9 | If I were to pull out the penis before ejaculation, my partner would think I was trying to avoid getting HIV. | **1** | **2** | **3** | **4** |
| WD10 | If I were to pull out the penis before ejaculation, my partner would think I was trying to avoid giving HIV. | **1** | **2** | **3** | **4** |
| WD11 | If I were to pull out the penis, my partner would think that I am unable to complete the job. | **1** | **2** | **3** | **4** |
| WD12 | If I were to pull out the penis, my partner would laugh at me | **1** | **2** | **3** | **4** |
| WD13 | If I were to pull out the penis, my partner would think I was a strong man | **1** | **2** | **3** | **4** |
| WD14 | If I were to pull out the penis, my partner would think I have good self control. | **1** | **2** | **3** | **4** |
| WD15 | If I were to pull out the penis, my partner would think I was sexually inexperienced. | **1** | **2** | **3** | **4** |

|  | | | | | | **CODE** |
| --- | --- | --- | --- | --- | --- | --- |
| RIS16 | **In the last 3 months, how many of your sex partner(s) were male?** | Enter no. of male partners 🡪 | | | |  |
| RISMCMFRQ | **In the last 3 months, how frequently did you use condoms with your male partners?** | 0= Never  1= Sometimes (less than half the time)  2= Frequently (more than half the time)  3= Always  4= Not asked (no male partners) | | | |  |
| RIS16a | **Of these ___ partners how many were new partners, that is, someone you never had sex with before?** | Enter no. of new male partners 🡪 | | | |  |
| RIS16b | **How frequently did you use condoms when having sex with these ____ new male partners?** | 0= Never  1= Sometimes (less than half the time)  2= Frequently (more than half the time)  3= Always  4= Not asked (no new male partners) | | | |  |
| RIS17 | **In the last 3 months, how many of your sex partners were female?** | Enter no. of female partners 🡪 | | | |  |
| RISMCFFRQ | **In the last 3 months, how frequently did you use condoms with your female partners?** | 0= Never  1= Sometimes (less than half the time)  2= Frequently (more than half the time)  3= Always  4= Not asked (no female partners) | | | |  |
| RIS17a | **Of these ___ partners how many were new partners, that is, someone you never had sex with before?** | Enter no. of new female partners 🡪 | | | |  |
| RIS17b | **How frequently did you use condoms when having sex with these ____ new female partners?** | 0= Never  1= Sometimes (less than half the time)  2= Frequently (more than half the time)  3= Always  4= Not asked (no new female partners) | | | |  |
| RIS28 | **How many of these ____male and female partners where HIV infected?** | 0= No  1= Yes 🡪 if yes, go to next Q  88= Don’t know | | | |  |
| PAR2 | **Have any of this/these HIV-infected partner/s taken any ARVs (HIV medication) in the last 3 months?** | 0= None  1= Some  2= All  88= Don’t know | | | |  |
| RIS28b | **How frequently did you use condoms when having sex with these HIV infected partners?** | 0= Never  1= Sometimes (less than half the time)  2= Frequently (more than half the time)  3= Always  4= Not asked (no HIV-infected partners) | | | |  |
| RIS29 | **Of these ___ sex partners how many did you *receive* any money, gifts or help from in exchange for having sex with them?**  (by gifts or help I mean payment of fees, transport, food, phone minute, clothes, money or anything else payment may or may not have been at the same time as the sex.) | | | | Code number 🡪 |  |
| RIS210 | **Of these ___ sex partners how many did you *give* any money, gifts or help from in exchange for having sex with them?**  (by gifts or help I mean payment of fees, transport, food, phone minute, clothes, money or anything else payment may or may not have been at the same time as the sex.) | | | | Code number 🡪 |  |
| RIS211 | **In the last 3 months, have you been forced to have sex against your will?** | | | 0= No  1= Yes 🡪 refer to supervisor for f/up | |  |
| RIS212 | **In the last 3 months, have you travelled or stayed away from home frequently?** (more than 3 nights week on average per week) | | | 0= No  1= Yes | |  |
| RIS213 | **In the last 3 months have you engaged in group sex, that is, sex with more than one person at the same time?** | | | 0= No  1= Yes, women only  2= yes, men only  3= Yes, men and women  4= Not asked | |  |
| RIS214 | **In the last 3 months have you had receptive (bottom) anal sex, that is, where you sex partner put his penis in your anus?** | | 0= No  1= Yes  2= Not asked | | |  |
| RIS214a | **How frequently did you use condoms when having receptive (bottom) anal sex, that is when your sex partner put his penis in your anus?** | | 0= Never  1= Sometimes (less than half the time)  2= Frequently (more than half the time)  3= Always  4= Not asked (no anal sex) | | |  |
| RIS215 | **MEN ONLY: In the last 3 months have you had insertive (top) anal sex, that is, put your penis in your sex partner’s anus?** | | 0= No  1= Yes  2= Not asked | | |  |
| RIS215a | **MEN ONLY: How frequently did you use condom when having insertive (top) analy sex, that is, when you put your penis in your sex partner’s anus?** | | 0= Never  1= Sometimes (less than half the time)  2= Frequently (more than half the time)  3= Always  4= Not asked (no anal sex) | | |  |

**COLLECT FROM ALL: VITAL SIGNS (**Nurse, enrolled nurse, doctor or clinician to take vitals)

|  | | | | | CODE | | | | | | | | | |
| --- | --- | --- | --- | --- | --- | --- | --- | --- | --- | --- | --- | --- | --- | --- |
| V0 | **Who took these vitals?** | 0=Enrolled Nurse  1= Professional Nurse  2= Doctor  3= Other | | |  | | | | | | | | | |
|  | | | | | | | | | | | | | | |
| v1bp  PEX11 | **Blood pressure**  record mm/Hg🡪 | |  |  | |  | / | |  | |  | |  | |
|  | | | | | | | | | | | | | | |
| v2p  PEX12 | **Pulse**  record beats per minute🡪 | | | | | | | |  | |  |  | | |
|  | | | | | | | | | | | | | | |
| vrr  PEX13 | **Respiration Rate**  record breaths per minute 🡪 | | | | | | | | |  | |  | | |
|  | | | | | | | | | | | | | | |
| VBT  PEX14 | **Body Temperature** record to 1 decimal place in degrees Celcius ( XX.X) 🡪 | | | | | | |  |  | | **.** |  | |  |
|  | | | | | | | | | | | | | | |
| V5 W  PEX15 | **Weight** record in kg🡪 | | | | | | | |  | |  |  | | |
|  | | | | | | | | | | | | | | |
| V6H  PEX16 | **Height**  record in cm🡪 | | | | | | | |  | |  |  | | |

_________________ **End time: _______:______**

**Staff code hh mm**

**Date: ___ ___ /___ ___ ___ / ___ ___ ___ ___**

**DD / MON / Y Y Y Y**
